# Supplementary material for: MetaRibo-Seq measures translation in microbiomes
Source: Nat Commun. 2020 Jun 29;11:3268. doi: 10.1038/s41467-020-17081-z (PMC7324362; doi:10.1038/s41467-020-17081-z)
Supplement: Supplementary file 10 — Supplementary Data 7 [file 41467_2020_17081_MOESM10_ESM.zip › File2/Confidence_High_Taxonomy/378539_out.krona.html]

Javascript must be enabled to view this page.

members
magnitude
magnitudeUnassigned
count
unassigned
taxon
rank

378539\_out

28

superkingdom
2
28

28
1239
phylum

class
28
186801

186802
28
order


SRS011134\_contig\_number\_49988SRS012849\_contig\_number\_18061SRS013951\_contig\_number\_2501SRS013965\_contig\_number\_42129SRS014235\_contig\_number\_47437SRS015065\_contig\_number\_16188SRS015663\_contig\_number\_9255SRS018133\_contig\_number\_14159SRS019787\_contig\_number\_13172SRS022609\_contig\_number\_52861SRS023914\_contig\_number\_27694SRS024663\_contig\_number\_15695SRS045826\_contig\_number\_contig-100\_52.144675SRS049959\_contig\_number\_49894SRS055017\_contig\_number\_24179SRS056273\_contig\_number\_6513SRS063518\_contig\_number\_39864SRS075078\_contig\_number\_29969SRS098827\_contig\_number\_13594SRS1041031\_contig\_number\_27337SRS1041140\_contig\_number\_22216SRS104912\_contig\_number\_3847SRS142503\_contig\_number\_56592SRS143523\_contig\_number\_17261SRS144506\_contig\_number\_16272SRS144537\_contig\_number\_18198SRS148721\_contig\_number\_53016SRS893383\_contig\_number\_35992
species
28
1897049
